# Epigenetic silencing of ZNF132 mediated by methylation-sensitive Sp1 binding promotes cancer progression in esophageal squamous cell carcinoma

**DOI:** 10.1038/s41419-018-1236-z

**Published:** 2018-12-18

**Authors:** Dong Jiang, Zhenglei He, Chenji Wang, Yinghui Zhou, Fang Li, Weilin Pu, Xueqing Zhang, Xulong Feng, Meng Zhang, Xinyue Yecheng, Yunyun Xu, Li Jin, Shicheng Guo, Jiucun Wang, Minghua Wang

**Affiliations:** 1 Department of Biochemistry and Molecular Biology, Medical College, Soochow University, Suzhou, Jiangsu China; 2 Department of Cardio-Thoracic Surgery, The First Affiliated Hospital of Soochow University, Suzhou, Jiangsu China; 3 Department of Human Anatomy, Histology and Embryology, Medical College, Soochow University, Suzhou, Jiangsu China; 4 State Key Laboratory of Genetic Engineering, Department of Anthropology and Human Genetics, School of Life Sciences, Fudan University, Shanghai, China; 5 Human Phenome Institute, Fudan University, Shanghai, China; 6 Institute for Pediatric Research, Affiliated Children’s Hospital, Soochow University, Suzhou, Jiangsu China; 7 Center for Human Genetics, Marshfield Clinic Research Institute, Marshfield, WI USA

## Abstract

Epigenetic alteration of tumor suppression gene is one of the most significant indicators in human esophageal squamous cell carcinoma (ESCC). In this study, we identified a novel ESCC hypermethylation biomarker *ZNF132* by integrative computational analysis to comprehensive genome-wide DNA methylation microarray dataset. We validated the hypermethylation status of *ZNF132* in 91 Chinese Han ESCC patients and adjacent normal tissues with methylation target bisulfite sequencing (MTBS) assay. Meanwhile, *ZNF132* gene silencing mediated by hypermethylation was confirmed in both solid tissues and cancer cell lines. What is more, we found that in vitro overexpression of *ZNF132* in ESCC cells could significantly reduce the abilities of the cell in growth, migration and invasion, and tumorigenicity of cells in a nude mouse model. We validated the Sp1-binding site in the *ZNF132* promoter region with chromatin immunoprecipitation assay and demonstrated that the hypermethylation status could reduce the Sp1 transcript factor activity. Our results suggest that *ZNF132* plays an important role in the development of ESCC as a tumor suppressor gene and support the underlying mechanism caused by the DNA hypermethylation-mediated Sp1-binding decay and gene silencing.

## Introduction

Esophageal cancer (EC) ranks eighth in most common cancers and sixth in cancer-related mortality worldwide^1,2^. For the past several decades, the incidence of and estimated deaths due to esophageal cancers have been increasing continuously. Based on GLOBOCAN worldwide estimates of cancer incidence and mortality produced by the International Agency for Research on Cancer, 455,800 new esophageal cancer cases and 400,200 deaths occurred in 2012 worldwide^3,4^. The 5-year survival rate of esophageal cancer remains poor despite the advances in clinical oncology. Esophageal cancer consists mainly of two subtypes, esophageal squamous cell carcinoma (ESCC) and esophageal adenocarcinoma (EAC), each with distinct pathologies and etiologies^5^. While EAC predominates in North America^6,7^, the majority of esophageal cancer cases worldwide are ESCC, which has a high prevalence in East Asia, Eastern and Southern Africa, and Southern Europe^8,9^. ESCC accounts for more than 90% of esophageal cancers in China nowadays^10^. In the past several years, genetic research on esophageal carcinoma have received several important achievements. A genome-wide association study identified serials of ESCC susceptibility genes^11–14^, such as *PLCE1* and *C20orf54*. Meanwhile, ESCC-associated microRNA-single-nucleotide polymorphisms^15,16^, copy number variations^17^, and somatic mutations^18^ have been widely identified. However, the mortality rate of ESCC was not be effectively controlled even with this achievement. Considering the characteristics of highly invasive, metastatic, and poor prognosis, there is an urgent need for identifying diagnostic and prognostic biomarkers for ESCC.

DNA methylation is one of the most intensively studied epigenetic modifications and it is involved in different biological processes, including development, gene expression regulation, and imprinting^19^. Multiple studies have confirmed that global hypomethylation induces genomic instability leading to cell transformation, and hypermethylation of promoter regions of the tumor suppressor genes facilitates tumorigenesis^20^. Previous studies have shown that a broad range of genes are silenced by DNA hypermethylation in different cancer types^21^. The study of specific DNA methylation has translational potential in the management of ESCC patients, and hypermethylated promoters may serve as candidate biomarkers. Moreover, DNA methylation is reversible, which makes it very interesting for therapy approaches^22^. We have previously screened TCGA (The Cancer Genome Atlas) database for aberrant epigenetic changes in ESCC. The results were validated with DNA methylation datasets from Gene Expression Omnibus, peripheral blood mononuclear cells, and peripheral blood leukocytes of healthy controls. Hypermethylation of several candidate genes was identified^23,24^ and one of them is *ZNF132*, which belongs to C2H2 zinc finger protein family. It is located at chromosome 19q13.4, which is usually deleted in thyroid adenomas^25^. *ZNF132* has 18 C2H2 zinc finger motifs according to its predicted structure. The zinc finger protein family has been shown to participate in biological processes such as development and differentiation. Recent studies have also suggested that zinc finger proteins play a role in cancer progression^26^. There are not many studies on the biological function of *ZNF132*; however, decreased expression of *ZNF132* has been reported in prostate cancer and is associated with aggressive prostate cancers^27^.

To determine the role of *ZNF132* and its potential value as a biomarker in ESCC, we studied the methylation status of the *ZNF132* promoter and the expression level of *ZNF132* in ESCC tumors and adjacent normal tissues. The relationship between methylation status and expression of *ZNF132* was investigated in vitro in EC cell lines with or without demethylation drugs. Furthermore, we tested the effect of *ZNF132* expression on proliferation, migration, invasion, and apoptosis of ESCC cells in vitro. The effect of *ZNF132* overexpression on the tumorigenicity of ESCC cell line was also tested in a nude mouse model. Finally, the mechanism of association of promoter hypermethylation and expression of *ZNF132* was explored.

## Materials and methods

### Human tissues, cell lines, transfection, and drug treatment

ESCC samples and their paired adjacent control tissues were obtained for validation study from the First Affiliated Hospital of Soochow University and Fourth Military Medical University between the years of 2011 and 2015. Ec-109 and CaEs-17 cells were obtained from the Shanghai Institute for Biological Sciences and grown in RPMI-1640 culture medium supplemented with 10% fetal bovine serum (GIBCO^®^, Invitrogen™, Auckland, New Zealand), penicillin (100U/ml), and streptomycin (100 μg/ml). HEK293T cells were maintained in Dulbecco's modification of Eagle's medium culture medium containing 10% fetal bovine serum (Gibco^®^, Invitrogen™, Auckland, New Zealand) that was supplemented with penicillin (100U/ml) and streptomycin (100 μg/ml). Cultured cells were grown at 37 °C in a humidified atmosphere of 5% CO_2_ and were passaged using pancreatic enzymes two or three times a week. HEK293T cells were co-transfected with the lentiviral vectors, 0.8 μg pSPAX2, 0.4 μg pMD2.G, and 1.2 μg pCD513B-*ZNF132* expression plasmid. One day before infection, CaEs-17 or Ec-109 cells were seeded in 6-well plates with a density of 2 × 10^5^ cells per well, and incubated at 37 °C in a humidified atmosphere of 5% CO_2_ overnight. 5-aza-2′-deoxycitidine (5-Aza) (Sigma-Aldrich, St. Louis, MO, USA) was used as a demethylating agent to treat cells. Drug treatment protocol was as previously described^28,29^.

### DNA methylation evaluated by MTBS

Methylation-targeted bisulfite-sequencing method (MTBS) was applied for the methylation profile investigation in this study. Non-CpG containing bisulfite sequencing primer was used for non-bias bisulfite-converted DNA replication with biocode for the sample identification and then followed by next-generation sequencing and the details could be found in our previous study^23,24^. Genomic DNA from ESCC tumor tissue and adjacent control tissue samples were extracted by AIIperp DNA/RNA Mini Kit (Qiagen, Duesseldorf, Germany) according to the manufacturer’s protocols. For methylation analysis, 500 ng genomic DNA was subjected to bisulfite conversion using the EpiTect Fast DNA Bisulfite Kit (Qiagen, Duesseldorf, Germany). Multiplex PCR was performed first with optimized non-CpG primer set combination (LINE-1, *ZNF132*, and ChrM). PCR amplicons were diluted and amplified using indexed primers and the products (170–270 bp) were separated by agarose electrophoresis and purified by QIAquick Gel Extraction Kit (Qiagen, Duesseldorf, Germany). Libraries from different samples were quantified and pooled together equally, and then sequenced with the Illumina Hiseq 2000 platform according to the manufacturer’s protocols. BSseeker2 software was utilized for reads mapping and methylation calling^30^. Samples with high missing rates (>30%) and CpG sites with high missing rates (>20%) were removed. Thirteen CpG sites located at the promoter of LINE-1 gene (hypomethylation in esophageal cancer tissues) and 11 CpG sites from mitochondrion DNA (ChrM) were also sequenced as positive and negative controls, respectively. Primers used are listed in Supplementary Table 1. Average methylation fraction (AMF) to each CpG loci or whole promoters was calculated to make the differential methylation test which has been applied in our previous studies^23,24,31^.

### RNA extraction and quantitative real-time PCR

Total RNA was isolated by AIIperp DNA/RNA Mini Kit (Qiagen, Duesseldorf, Germany). First-strand complementary DNA (DNA) was synthesized from 1 μg total RNA with a High-Capacity cDNA Reverse Transcription Kit (Applied Biosystems, Foster City, CA, USA). Quantitative real-time PCR (q-PCR) was carried out with an Applied Biosystems 7900 Prism real-time PCR machine and SYBR Premix Ex Taq (Takara, Dalian, Japan), in accordance with the manufacturer’s instructions. Glyceraldehyde-3-phosphate dehydrogenase (GAPDH) was used as an internal reference. Quantitative real-time PCR primers used for *ZNF132* were listed in Supplementary Table 1. The target gene expression in test samples was normalized to the corresponding GAPDH level and was reported as the fold difference relative to the GAPDH gene expression.

### Cell proliferation assay

Ec-109 and CaEs-17 cells both treated by the plasmid pCD513B-*ZNF132* and pCD513B for 48 h were incubated for 0, 24, 48, 72, 96, and 120 h in 96-well plates with 1000 cells/well. Then, 10 µl CCK-8 (Tianjin Biolite Biotechnologies, Tianjin, China) was added to each well for 3 h, followed by light absorbance measurement at a wavelength of 450 nm.

### Transwell assays for cell migration and invasion

The suspension of the Ec-109 cells treated by the plasmid pCD513B-*ZNF132* and pCD513B was prepared in a non-serum medium with a density of 2 × 10^5^ cells/ml, 200 μl suspension of this kind was plated on the top side of a polycarbonate Transwell filter coated with Matrigel in the upper chamber of the BioCoat™ Invasion Chambers (BD, Bedford, MA, USA) and incubated at 37 °C for 24 h, and 500 μl culture medium was added in each well, then the cells inside the upper chamber were removed with cotton swabs, invaded cells on the lower membrane surface were fixed in 4% paraformaldehyde, stained with crystal violet, and counted (five random fields per well at ×100 magnification).

### Wound-healing assay

CaEs-17 and Ec-109 cells treated by the plasmid pCD513B-*ZNF132* and pCD513B were seeded into 6-well plates at a density of 2 × 10^5^ cells/well. When they had nearly reached confluency, a wound was created by manually scraping the cell monolayer with a 10 μl pipette tip and then cells were washed twice with 1× phosphate-buffered saline (PBS). Some cells were harvested here (time, 0 h), while others were maintained for 96 h in the culture medium, and pictures were taken under the inverted microscope.

### Assessment of apoptosis

Ec-109 cells treated by the plasmid pCD513B-*ZNF132* and pCD513B were seeded in 6-well plates with a density of 2 × 10^5^ cells per well for 48 h. Cells were suspended with trypsin, harvested, and stained with Annexin V-PE/7-AAD. Afterwards, the cells were analyzed by a flow cytometer (FACS Calibur; Becton-Dickinson, Mountain View, CA, USA).

### Dual-luciferase reporter gene assay

Dual-luciferase reporter gene assay was performed as previously described^32^. The fragment in the *ZNF132* promoter region (chr19: 58,951,628–58,951,928, hg19) was cloned to pGL3-Basic vector (Promega, Madison, WI, USA) or pCpGL-Basic vector (the pCpGL-Basic vector was a kind gift from M. Rehli, University Hospital Regensburg) to make a reporter construct. The construct inserts were verified by sequencing. To confirm the participation of Sp1, HEK293T cells plated on 24-well plates were transfected with 0–1.6 μg Sp1 expression vector and 200 ng pGL3-*ZNF132* or 200 ng methylated pGL3-*ZNF132* by Lipofectamine 2000 (Life Technologies, Carlsbad, CA, USA). In each transfection, 5 ng of pRL-SV40 vector (Promega, Madison, WI) was used to correct the transfection efficiency. To determine whether methylation of Sp1-binding site alters the transcriptional activity of the *ZNF132* promoter by derecruiting Sp1, HEK293T cells plated on 24-well plates were transfected with 0, 300 ng Sp1 expression vector and 200 ng pCpGL-*ZNF132* or 200 ng methylated pCpGL-*ZNF132* by Lipofectamine 2000. The methylated reporter construct were methylated in vitro using M.SssI (CpG) Methyltransferase as recommended by the manufacturer’s instructions. In each transfection, 5 ng of pRL-SV40 vector was used to correct the transfection efficiency. The luciferase activity was measured with the Dual-Luciferase Reporter Assay System (Promega, Madison, WI, USA). Promoter activities were expressed as the ratio of Firefly luciferase to Renilla luciferase activities. Transfection experiments were repeated at least three times.

### ChIP assay

Chromatin immunoprecipitation (ChIP) assay was performed as previously described^33,34^. Briefly, Ec-109 cells were transfected with p3 × flag-Sp1 or p3 × flag-cmv-10 vectors. Transfected for 48 h, cells were crosslinked with 1% formaldehyde for 10 min at room temperature. Chromatin was sheared using Bioruptor^®^ Plus sonication device (Diagenode, Belgium) to obtain DNA fragments of about 400−600 bp. The anti-FLAG affinity gel (Bimake, Product ID B23101) was used to pull down the flag-tagged proteins. The chromatin was then de-cross-linked at 65 °C overnight with proteinase K (New England Biolabs, Ipswich, MA, USA). DNA was purified using the MinElute PCR Purification Kit. Purified ChIP DNA was subjected to PCR, which amplified the *ZNF132* promoter region encompassing the putative Sp1-binding site. Specific ChIP primers used for PCR were: 5′-CAGCCGAGGAGACAGGCACTT-3′ (forward) and 5′-CCCAGGGAGCCTCCAAGATT-3′ (reverse).

### DNA pull-down assay

The DNA pull-down assay was performed according to a previous report^35–37^. The promoter region of *ZNF132* gene was amplified by PCR using 5′-biotin-labeled primer. The primer sequences were as follows: forward primer, CACTTCCGGGCGGAGTGTAAGA; reverse primer, TTCCGTCCCTCGCCTGACAAC. The methylated biotinylated double-stranded DNA were methylated in vitro using M.SssI (CpG) Methyltransferase as recommended by the manufacturer’s instructions (New England Biolabs, Beverly, MA, USA). Cell lysates were extracted from HEK293T cells transiently transfected with p3 × flag-Sp1 vector. Cell lysis (400 μg) and methylated or unmethylated probe (0.5 pmol) were incubated for 2 hours at 4 °C in the presence of streptavidin-agarose beads (Roche, USA). The binding reaction system was 10 mM Tris-HCl (pH 7.5), 1 mM EDTA and 100 mM NaCl. Due to the affinity of the streptavidin magnetic beads for biotinylated probe, the DNA–protein complexes were pulled down with streptavidin–agarose beads by centrifugation at 200 × *g* for 60 s. The pulled-down complex was washed three times with 1 ml ice-cold TBS buffer (20 mM Tris, 150 mM NaCl), separated on a sodium dodecyl sulfate (SDS)-polyacrylamide gel, and analyzed by western blotting. The antibody used in this experiment was Sp1 antibody (1:1000, Cell Signaling Technology, Danvers, MA, USA). To quantify the strength of the DNA–protein complex, 20× molar non-biotinylated probe were added to the pull-down mixture as competitors for the biotinylated probe.

### Western blotting

Ec-109 and CaEs-17 cells were lysed, cellular proteins were separated by 10% SDS-polyacrylamide gel electrophoresis, and the resolved proteins were electroblotted onto a polyvinylidene difluoride membrane (Millipore, Billerica, MA, USA). Membranes were blocked for 1 h in Tris-buffered saline containing 10% nonfat dry milk and 0.1% Tween-20 and then probed at 37 °C for 1 h with rabbit anti-ZNF132 antibody (1:1000, Aviva Systems Biology, CA, USA) as the primary antibody. After rinsing with PBS, the membrane was treated with goat anti-rabbit immunoglobulin G-horseradish peroxidase (Boster, China) as the secondary antibody. Bound proteins were visualized with a Tanon 5200 chemiluminescence imaging system (Tanon, China). β-Actin was detected with an anti-β-actin rabbit antibody (1:1000; Santa Cruz Biotech, Santa Cruz, CA, USA) to demonstrate equal protein sample loading.

### Xenograft tumor mouse model

All animal experiments were approved by the Soochow University. Four-week-old male BALB/c nude mice (nu/nu; *n* = 7) (Soochow University Laboratory Animal Center, China) were anesthetized with an isoflurane/propylene glycol mixture, and Ec-109 stable cell lines with pCD513B-*ZNF132* or pCD513B were subcutaneously injected into each mouse (2.0 × 10^6^ cells in 200 μl PBS. The tumor sizes were assessed every 3 days by measuring two dimensions, and the tumor volumes were calculated as the volume = (tumor length) × (tumor width)^2^/2^38^. The tumors were collected and weighed 30 days.

### Statistical analysis

We tested the differential methylation of the overall CpG sites between cancer and normal tissues using Wilcoxon's rank-sum test. We evaluated 15 CpGs located in the promoter regions of ZNF132, and only the CpGs in the differential methylation region (DMR) were used for further diagnosis or prediction analysis, or other statistical analysis. Methylation and gene expression correlation in cancer samples were applied linear regression after log transforming to relative gene expression. Correlation analysis between DNA methylation and age, weight was applied with linear regression, while others, such as tumor node metastasis (TNM), cancer onset location, gender and smoking, as well as drinking, were applied with one-way analysis of variance (ANOVA). All statistical analyses were conducted using R 3.2.1. GraphPad Prism5 (GraphPad, San Diego, CA, USA) and R scripts were used to make the figures.

## Results

### Hypermethylation of *ZNF132* in esophageal squamous cell carcinoma

**Fig. 1:**
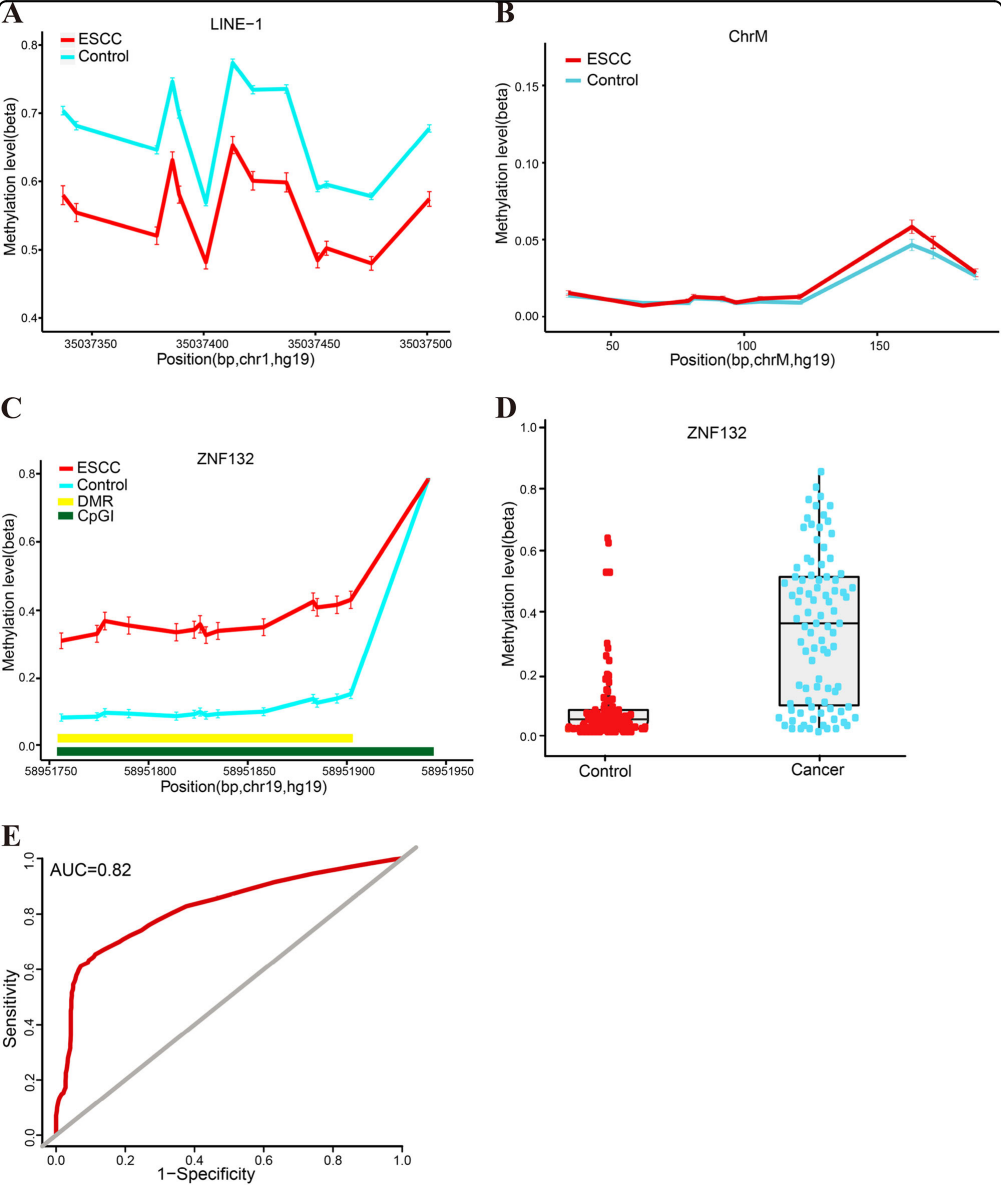
Hypermethylation of *ZNF132* in esophageal squamous cell carcinoma. **a** Median % methylation values of 13 CpG site of LINE-1. **b** Median % methylation of 11 CpG sites of ChrM. **c** Median % methylation in ESCC and adjacent control tissues of 15 CpG sites of the *ZNF132* promoter region (except the last CpG which is hypermethylated in both normal and cancer samples). DMR represents differentially methylated regions and CpGI represents CpG island. **d** The methylation of *ZNF132* in the 91 cases of ESCC and adjacent tissues (each point represents the absolute ratio of methylation in each tissue) **e** represents the overall ROC (receiver operating characterstics) curve, which was calculated through a logistic regression model, incorporating the mean methylation percentage of the five genomic regions as the variables, and with the adjustment for gender, age, smoking, and alcohol consumption

Methylation-targeted bisulfite-sequencing method (MTBS) was used to determine the methylation status in the promoter regions (15 CpG sites) of *ZNF132* in 91 ESCC and adjacent normal tissues from Han Chinese population (Table 1). We found that the methylation level of LINE-1 was significantly lower in cancer samples compared with normal tissues (overall *P* < 2.16 × 10^−8^), while technique negative control (ChrM) was absolutely low AMF (<0.03) in our samples (Fig. 1a, b), which are highly consistent with the previous studies^39,40^, demonstrating the reliability and robustness of our targeted bisulfite-sequencing method. We found that the methylation profile of *ZNF132* were significantly higher in ESCC tumors than that in adjacent control tissues (Fig. 1c, d). A significant DMR including 14 CpG sites was identified in the core promoter region of *ZNF132* (overall *P* value = 2.2 × 10^−16^ and Table 2). In addition, we found that the promoter methylation was slightly different between the different locations of cancer onset (*P* = 0.07, ANOVA): samples from the upper part of the esophagus had higher methylation levels (AMF = 0.53) compared with the middle (AMF = 0.39) and lower (AMF = 0.27) parts. Meanwhile, although TNM is not significantly associated with *ZNF132* methylation (*P* = 0.71), we identified a significant association with the number of nearby lymph nodes (*N* value in TNM stage). We did not detect other significant correlation between *ZNF132* methylation and age (*β* = 0.002, *P* = 0.395), gender (*P* = 0.28), drinking (*P* = 0.54), smoking (*P* = 0.78), and weight (*P* = 0.34). We also examined the prediction performance of *ZNF132* hypermethylation in ESCC diagnosis. The prediction model with logistic regression shows the sensitivity (70.8%), specificity (80.6%), and area under curve (AUC = 0.82) with the adjustment of ESCC main risk factors, including age, gender, smoking, and alcohol consumption, demonstrating that hypermethylation of *ZNF132* could be taken as a strong diagnostic biomarker for ESCC (Table 2 and Fig. 1e).

**Table 1:** Clinical characteristics of the study population

Characteristics	*N* = 91	%
Age (years)		
<60	30	33
≥60	61	67
Age(mean ± SD)	63.20 ± 8.17	
Gender (*n*)		
Male	66	72.5
Female	25	27.5
Drinking^a^		
Yes	32	35.2
No	56	61.5
Unknown	3	3.3
Smoking^b^		
Yes	56	61.5
No	35	38.5
T stage^c^		
T1	1	1.1
T2	12	13.2
T3	72	79.1
T4	4	4.4
Unknown	2	2.2
N stage^c^		
N0	42	46.2
N1	37	40.7
N2	7	7.7
N3	3	3.3
Unknown	2	2.2
M stage^c^		
M0	88	96.7
M1	1	1.1
Unknown	2	2.2

*ESCC* esophageal squamous cell carcinoma

^a^Yes represents individuals who presently consume or formerly consumed alcoholic beverages

^b^Yes represents the former and current smokers

^c^TNM stages were assessed by the seventh edition of the TNM classification criteria

**Table 2:** The methylation of *ZNF132* gene and control gene in ESCC

Gene name	Mean (case)	Mean (control)	*P* value^a^	*P* value^b^	Sensitivity	Specificity	Area Under the curve (AUC)
*ZNF132*	0.36	0.11	2.2 × 10^−16^	2.2 × 10^−16^	70.8%	80.6%	0.82
*LINE-1*	0.55	0.73	2.16 × 10^−8^				
*ChrM*	0.03	0.02	2.59 × 10^−1^				

^a^*P* value is calculated through the Wilcoxon's rank-sum test followed by FDR (false discovery rate) adjustment for multiple correction

^b^*P* value determined by logistic regression

### Regulation of *ZNF132* expression by methylation of its promoter in ESCC patients and esophagus cancer cell lines

**Fig. 2:**
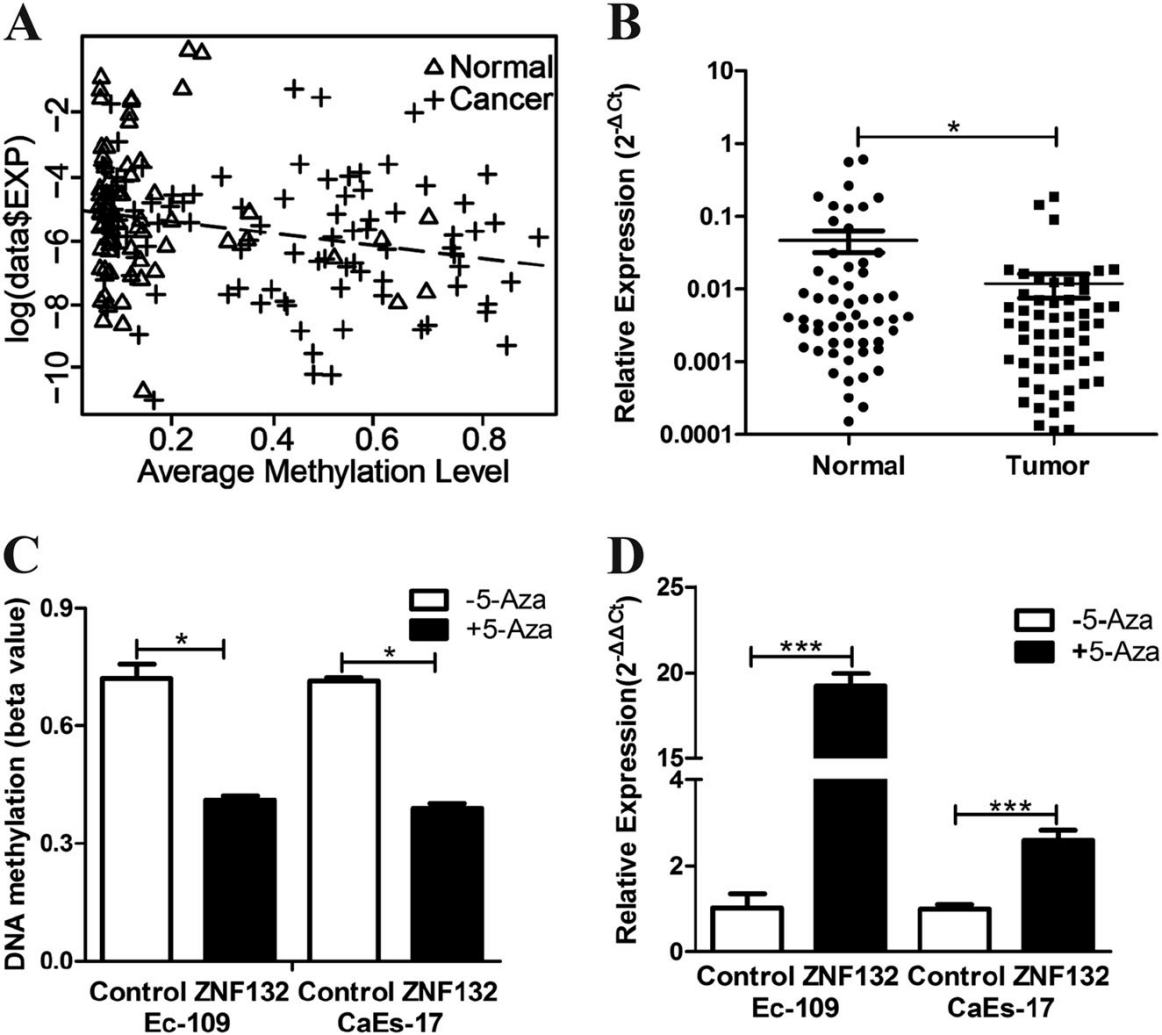
Methylation status and gene expression of *ZNF132* in ESCC patients and esophageal cancer cell lines. **a** Methylation and gene expression correlation in clinical samples. *Y*-axis is log-transferred to relative expression similar to that in Fig. 4a; *x*-axis represents the average methylation level. Dot line indicates the linear regression line. **b** Expression of *ZNF132* measured by q-PCR in ESCC tissues was significantly lower than that in adjacent tissues. **c** Methylation of *ZNF132* in Ec-109 and CaEs-17 cells was significantly reduced after 5-Aza treatment. **d** Expression of *ZNF132* measured by q-PCR significantly increased after 5-Aza treatment. Data are presented as the mean ± S.D. of three independent experiments. *P < 0.05, ***P < 0.001

As methylation of gene promoter regions is a well-known gene expression regulation mechanism, we first examined the expression of *ZNF132* in 91 pairs of tumor and adjacent control tissues from ESCC patients. Quantitative real-time PCR was used to evaluate the expression level of *ZNF132* in the samples. The results demonstrated a significantly higher level of *ZNF132* expression in adjacent control tissues than that in ESCC tissues (Fig. 2b). The expression-methylation regression analysis in clinical samples shows that gene expression of *ZNF132* was significantly negatively correlated with DNA methylation level in cancer clinical samples (*P* = 0.00284) (Fig. 2a), indicating that the expression profile of *ZNF132* in ESCC tissues was altered as a consequence of its promoter hypermethylation in ESCC patients. To confirm the relation between *ZNF132* methylation and its expression observed in ESCC patient tissues, two esophagus cancer cell lines (Ec-109, CaEs-17) were treated with demethylation reagent 5-Aza (5-aza-2′-deoxycytidine). The*ZNF132* promoter methylation levels of two lines decreased significantly after treatment (Fig. 2c), and at the same time, *ZNF132* expression level significantly increased (Fig. 2d). The results clearly established that the methylation status of *ZNF132* negatively regulates its expression. The fact that epigenetic treatment modulates *ZNF132* expression shows its potential as an epigenetic cancer therapy.

### Effects of ZNF132 on biological functions such as proliferation, apoptosis, and migration of ESCC cells

**Fig. 3:**
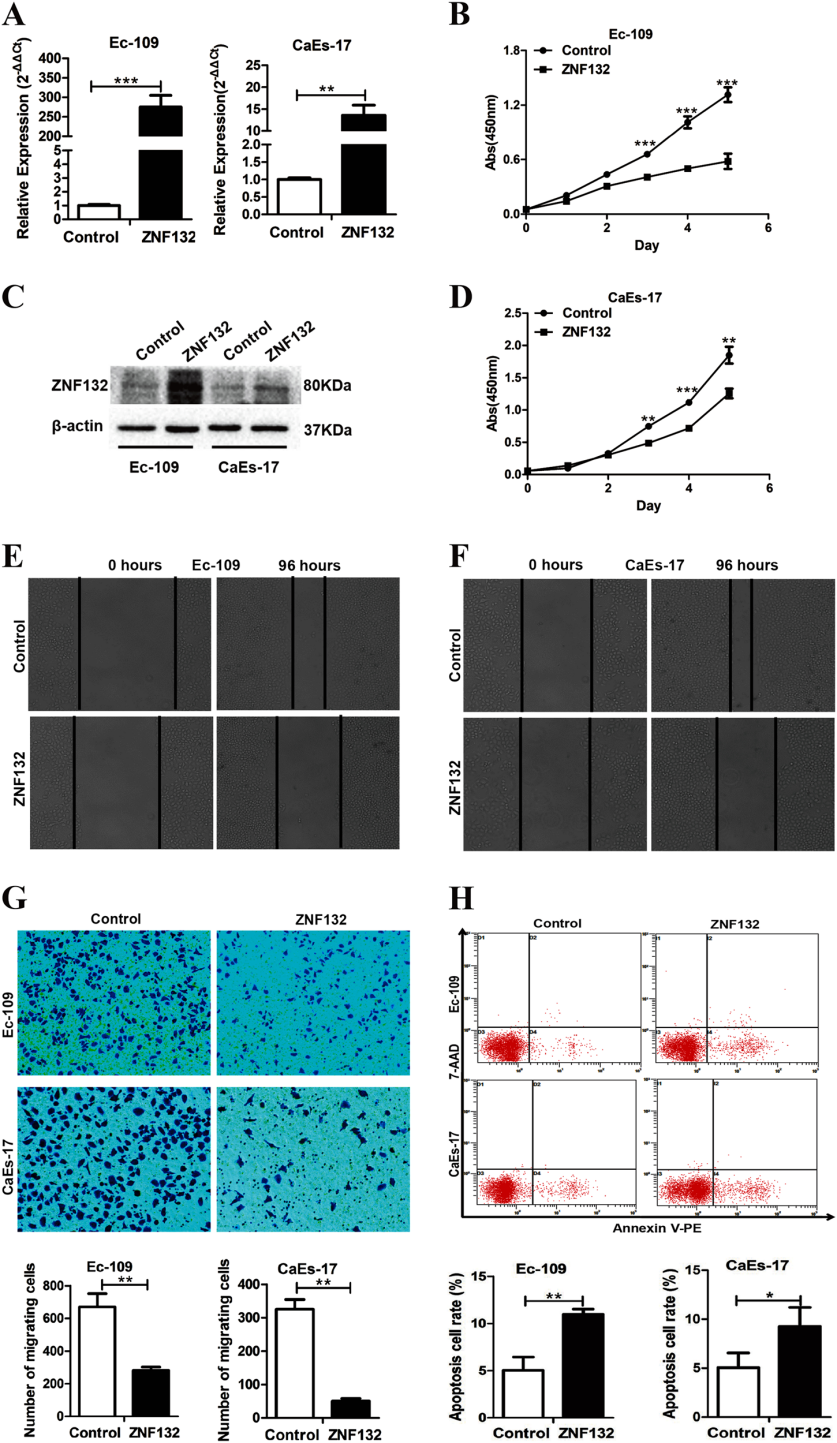
The effects of high expression *ZNF132* on characteristics of esophageal cancer cell lines in vitro. **a**, **c** Expression of *ZNF132* measured by q-PCR and western blotting in Ec-109 and CaEs-17 cells. **b**, **d** Effect of ZNF132 gene on the proliferation of esophageal cancer cells by cell proliferation assay. Light absorbance measurement at a wavelength of 450 nm was recorded to show that upregulation of *ZNF132* gene could inhibit the proliferation of CaEs-17 and Ec-109 cells. **e**, **f** In vitro scratch healing experiment showed that high expression of *ZNF132* in Ec-109 and CaEs-17 cells significantly inhibits the ability of the cells to heal. **g** The upregulation of *ZNF132* gene in Ec-109 and CaEs-17 cells reduced cell migration ability in a transwell assay. **h** Flow cytometry demonstrates that upregulation of *ZNF132* in CaEs-17 and Ec-109 cells could significantly increase the cell apoptosis rate. Data are presented as the mean ± SD. *P < 0.05, **P < 0.01, ***P < 0.001

To determine the effects of *ZNF132* protein on characteristics of ESCC cells, we constructed pCD513B-*ZNF132* plasmid and used empty pCD513B as a control. Expression of *ZNF132* measured by q-PCR and western blotting in Ec-109 and CaEs-17 cells (Fig. 3a, c). The effects of high expression of *ZNF132* were then tested on the abilities of the cells in growth, migration, invasion, and apoptosis. Cell growth of pCD513B-*ZNF132* was significantly slower than pCD513B cells since the third day (Fig. 3b, d). In vitro scratch healing experiment showed that high expression of *ZNF132* in Ec-109 and CaEs-17 cells significantly inhibits the cell healing ability (Fig. 3e, f). The abilities of the cells in migration were also negatively affected by the presence of pCD513B-*ZNF132* (Fig. 3g). These results indicate that *ZNF132* plays an inhibitory role in the growth, migration, and invasion of esophageal squamous carcinoma cells. Besides the abilities of the cells in growth, migration, and invasion, abnormal pattern of tumor cell apoptosis also plays a role in tumor growth and metastasis. The percentage of apoptotic Ec-109 and CaEs-17 cells significantly increased by high expression of *ZNF132* (Fig. 3h). The results show that higher expression of *ZNF132* greatly reduced tumorigenicity of ESCC cells in vitro.

### Reduction of tumorigenicity of ESCC cells by enforcing *ZNF132* expression in the in vivo xenograft model

**Fig. 4:**
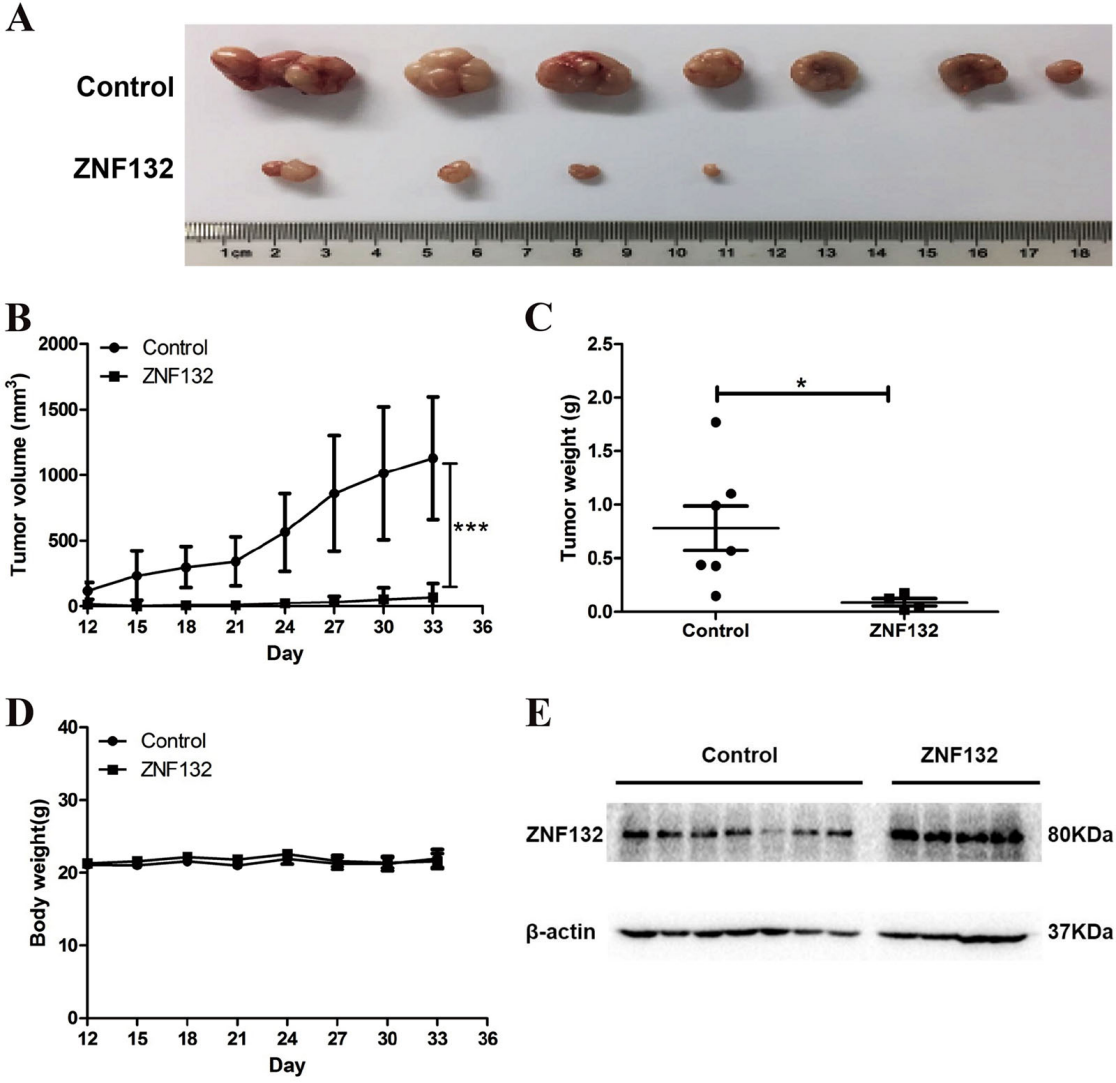
Overexpression of *ZNF132* inhibits the growth of human esophageal squamous cell carcinoma in vivo in a mouse xenograft model. **a** Small tumor volume with pCD513B-*ZNF132* transection. Ec-109 cells transfected with either the plasmid pCD513B-*ZNF132* or pCD513B were inoculated into the BALB/c nude mice. Tumor volume and mouse body weight were measured throughout the procedure. When the tumor reached the expected standard, the mice were sacrificed and the tumor was dissected, recorded, and the body wet weight of the tumor was measured. **b**, **c** It was evident from tumor volume (**b**) and tumor wet weight (**c**) that the overexpression of *ZNF132* gene could significantly inhibit the tumorigenic ability of Ec-109 cells. *P < 0.05, ***P < 0.001. **d** Body weight in the experimental group and the control group during the whole experiment. **e** Western blotting results to show that *ZNF132* was expressed in a subcutaneous injection of *ZNF132* stable cell line

To investigate whether *ZNF132* gene functions as a tumor suppressor also in vivo, we established the xenograft model. Ec-109 cells transfected with either the plasmid pCD513B-*ZNF132* or pCD513B were inoculated into the BALB/c nude mice. On day 33, the mice were sacrificed, and the volumes and wet weights of the tumors were measured individually. The tumor sizes of the pCD513B-*ZNF132* group were visually smaller than the pCD513B group (Fig. 4a). There were significant differences in tumor volume and wet weight between experiment and control groups (Fig. 4b, c), while there was no significant difference in body weight of mice between two groups during the experiment (Fig. 4d). We further examined the protein expression level of *ZNF132* in tumor tissues by Western blotting, and found that *ZNF132* was expressed in a subcutaneous injection of *ZNF132* stable cell line (Fig. 4e). No abnormal daily food and water consumption, and other adverse effects, such as mental state and hematuria, were observed. The xenograft study suggests that *ZNF132* plays a role as tumor suppressor gene in preventing ESCC in vivo.

### Methylation of Sp1-binding site inhibits *ZNF132* expression at the transcriptional level

**Fig. 5:**
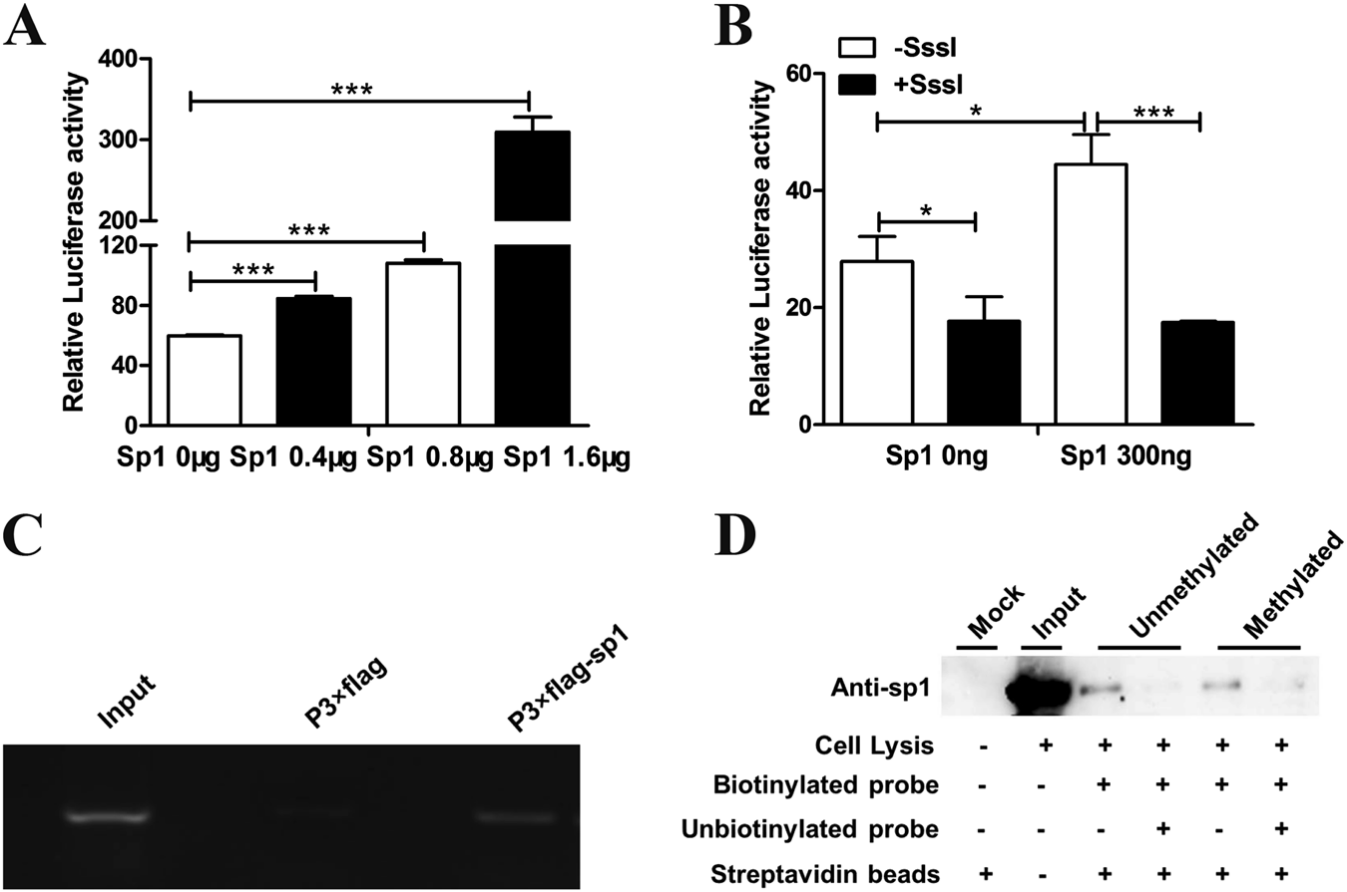
Hypermethylation of transcriptional activator Sp1-binding site in the *ZNF132* promoter region leading to *ZNF132* gene silencing in esophageal cell line. **a** Hypermethylation of the *ZNF132* promoter region decreases the gene expression of ZNF132. Sp1 and *ZNF132* promoter regions were transferred into HEK293T cells to determine activity. Data are presented as the mean ± SD. ***P < 0.001. **b** Methylated luciferase reporter assay shows that methylated Sp1-binding site resulted in a decrease in luciferase activity compared to the unmethylated site. Data are presented as the mean ± SD. *P < 0.05, ***P < 0.001. **c** ChIP assay to show that Sp1 protein can bind to the *ZNF132* promoter region containing Sp1 site in in vitro cultured cells. **d** The protein molecular weights of Sp1 is 90 kDa. DNA pull-down assay to confirm the hypermethylated probe of this segment had significantly lower binding affinity to Sp1 than the unmethylated probe

In order to demonstrate the *ZNF132* gene regulation mechanism, we tried to investigate the transcriptional factors' binding status in the *ZNF132* promoter region. We found the 6-base-pair (GGGCGG) Sp1-binding motif located in the CpG island of *ZNF132* promoter region and its binding region was supported by ENCODE Transcription Factor ChIP-seq data (Supplementary Figure 1). We then tried to determine whether methylation of Sp1-binding site plays a role in *ZNF132* expression regulation. According to the published sequence of the *ZNF132* promoter, luciferase reporter constructs were generated and transiently transfected together with increasing doses of Sp1 expression vector into HEK293T cells. The results showed that the transcriptional activity of the *ZNF132* promoter was elevated with increasing doses of Sp1, suggesting that Sp1 may transcriptionally modulate *ZNF132* expression (Fig. 5a).

To determine whether methylation of Sp1-binding site alters the transcriptional activity of the *ZNF132* promoter by derecruiting Sp1, we generated unmethylated luciferase reporter constructs containing the unmethylated fragments (Sp1-binding sequence). The methylated reporter constructs were methylated in vitro using M.SssI (CpG) Methyltransferase. The results showed that the methylated Sp1-binding site dramatically led to a reduction of luciferase activity compared with the unmethylated one, suggesting that methylation of Sp1-binding site can inhibit *ZNF132* transcriptional expression by interfering with the recruitment of Sp1 to *ZNF132* promoter region (Fig. 5b).

To determine directly whether Sp1 binds to *ZNF132* promoter, ChIP assay was performed. Using ChIP DNA purified from cultured cells transfected with p3 × flag-Sp1 vector, the results of PCR, which amplified the *ZNF132* promoter region encompassing the putative Sp1-binding site, showed a clear band, while no such band was seen if the cells transfected with p3 × flag-cmv-10 vectors were used (Fig. 5c). This clearly demonstrates that Sp1 can bind to the *ZNF132* promoter region in live cells cultured in vitro.

The DNA pull-down assays was used to confirm that the transcriptional activator Sp1 could bind to the promoter region of *ZNF132* gene and that the methylation status of *ZNF132* promoter negatively affects the binding. DNA pull-down assays showed that the methylated Sp1-binding site probe had weaker binding ability with Sp1 proteins compared with the unmethylated Sp1-binding site probe (Fig. 5d).

Although DNA methylation is theoretically caused by harmful environmental and genetic variant exposure, such as smoking and drinking, as well as risk allele carrying, it might also be due to different environmental triggers because of contact with different environments. Since DNA methylation can be reversed by appropriate treatment, DNA methylation is considered to be a promising diagnosis and prognosis biomarker. Combined with our results described above, the results revealed, at least in part, the mechanisms underlying the association of hypermethylation of the *ZNF132* promoter region and ESCC. Methylation of Sp1-binding site prevents the transcriptional activator Sp1 from binding to *ZNF132* promoter, silencing *ZNF132* tumor suppressor gene.

## Discussion

ESCC is a complex disease caused by different aberrant changes such as epigenetic, genetic, and environmental interactions^41^. Since the worse prognosis, early and accurate diagnosis provides an important approach to decrease the mortality. In the past decades, DNA methylation has been demonstrated to be a promising early diagnostic biomarker for ESCC; however, only a limited number of DNA methylation markers for early detection, recurrence, and prognosis have been identified in ESCC^42,43^. Further, there are not many studies focusing on mechanisms under which epigenetic changes in tumor suppressor gene promoter regions lead to human ESCC initiation and progression.

In this study, we show that *ZNF132* gene is silenced in ESCC tumor tissues, but not in adjacent control tissues in paired tissue samples from ESCC patients. In ESCC tumor tissue, the *ZNF132* gene is hypermethylated in its promoter region. The epigenetic changes in *ZNF132* in ESCC patients samples have been determined by targeted bisulfite sequencing. Methylation status of the *ZNF132* promoter region is significantly higher in ESCC tissue than in adjacent control tissue. *ZNF132* expression at the RNA level, consistent with its methylation status, is significantly lower in ESCC cells, indicating possible tumor suppressor function of *ZNF132*. These results have led us to further explore the clinical value of hypermethylation of *ZNF132* promoter. Logistic regression analysis has revealed that hypermethylated *ZNF132* is strongly associated with ESCC after adjustment for age, sex, smoking, and alcohol consumption. The logistic regression model was also used to evaluate the prediction ability of hypermethylation status of *ZNF132* promoter. Analysis results, sensitivity, specificity, and AUC with adjustment for age, sex, smoking, and alcohol consumption, indicate the moderate prediction ability of the test. Taken together, *ZNF132* hypermethylation is an independent diagnostic factor together with other risk factors, such as age, gender, smoking, and drinking.

To our knowledge so far, there have never been any studies of *ZNF132* in ESCC, actually there is only one report on the role of *ZNF132* in human cancer, demonstrating the significant inverse correlation between methylation level of *ZNF132* and its protein expression in tissue samples from prostate cancer patients^27^. Consistent with our study in ESCC patients, their results also illustrate that *ZNF132* have the potential to be a new candidate methylation marker for prostate cancer. The role of methylation promotor and expression of *ZNF132* were analyzed in in vitro study with EC cell lines. Two EC cell lines showed significantly decreased *ZNF132* methylation accompanied by increased expression of *ZNF132* after treatment with 5-Aza demethylation reagent, demonstrating directly the inverse relationship between promoter methylation status and expression of *ZNF132* in ESCC. The results indicate the potential of demethylation drugs as a epigenetic cancer therapy. The function of *ZNF132* was then studied in ESCC lines. Overexpression of *ZNF132* in ESCC cells greatly reduced the abilities of cells in growth, migration, and invasion, and significantly increased apoptotic cell death illustrating in vitro the tumor suppression function of *ZNF132*. The effect of *ZNF132* overexpression was also studied in vivo with a nude mouse model. The tumorigenicity of EC cells with overexpressed *ZNF132* is significantly reduced, therefore confirming the above in vitro results. Our study is the first one to show both in vitro and in vivo the tumor suppression function of *ZNF132*, indicating the pathological importance of reducing *ZNF132* expression by hypermethylation of its promoter region. The underlying mechanism of the effect of methylation status of *ZNF132* promoter on its expression was explored. Sp1 is a zinc finger protein that belongs to the SP family of transcription factors. The canonical sequence of the Sp1-binding site is 5′-(G/T)GGGCGG(G/A)(G/A) containing GpC in the promoter region^44^. Binding of Sp1 to a target gene can be interrupted by DNA methylation, resulting in silencing of gene expression. Sp1 is a ubiquitous transcriptional activator that is involved in a variety of biological processes, including cell proliferation and progression^45^. However, the role of Sp1 in human cancer remains elusive. Sp1 is thought to be a promoter or repressor of cell proliferation and progression^46,47^. Methylation of the CG site of the promoter region of the gene can spatially interfere with the binding of transcription factors such as Sp1 to DNA, thereby inhibiting transcription. Moreover, “CCGG” methylation was sufficient to prevent transcriptional stimulation by Sp1^48^. CpG was in silico predicted to be harbored in Sp1-binding site at *ZNF132* promoter. It was the first study to demonstrate that Sp1 can bind to the promoter region of *ZNF132*, and that the methylated site could prevent Sp1 from binding to the promoter. The mechanism of promoter methylation effects on gene expression is very complicated. Our results, therefore, indicate that hypermethylation of the *ZNF132* promoter region can reduce the ability of Sp1 to bind its DNA recognition elements, potentially damaging transactivation. Moreover, our results imply that prevention of binding of Sp1 to the *ZNF132* promoter region by hypermethylation may be one of the mechanisms for reducing ZNF132 expression in ESCC.

In conclusion, our study for the first time demonstrated that *ZNF132* promoter is hypermethylated in ESCC tissues, but not in adjacent control tissues. The effects of the epigenetic change and expression of *ZNF132* on tumorigenicity of EC cell lines were investigated both in vitro and in vivo. Preventing Sp1 from binding to *ZNF132* promoter was shown to be at least one of the underlying mechanisms. Most importantly, the methylation status of *ZNF132* promoter in the tumor tissues of ESCC patients is an independent prognostic factor, and has the potential use as a biomarker useful in prognosis of ESCC.

## Supplementary information

Supplementary Figure 1

Supplementary Table 1
