# Supplementary material for: Epigenetic silencing of ZNF132 mediated by methylation-sensitive Sp1 binding promotes cancer progression in esophageal squamous cell carcinoma
Source: Cell Death Dis. 2018 Dec 18;10(1):1. doi: 10.1038/s41419-018-1236-z (PMC6315024; doi:10.1038/s41419-018-1236-z)
Supplement: Supplementary file 1 — Supplementary Figure 1 [file 41419_2018_1236_MOESM1_ESM.docx]

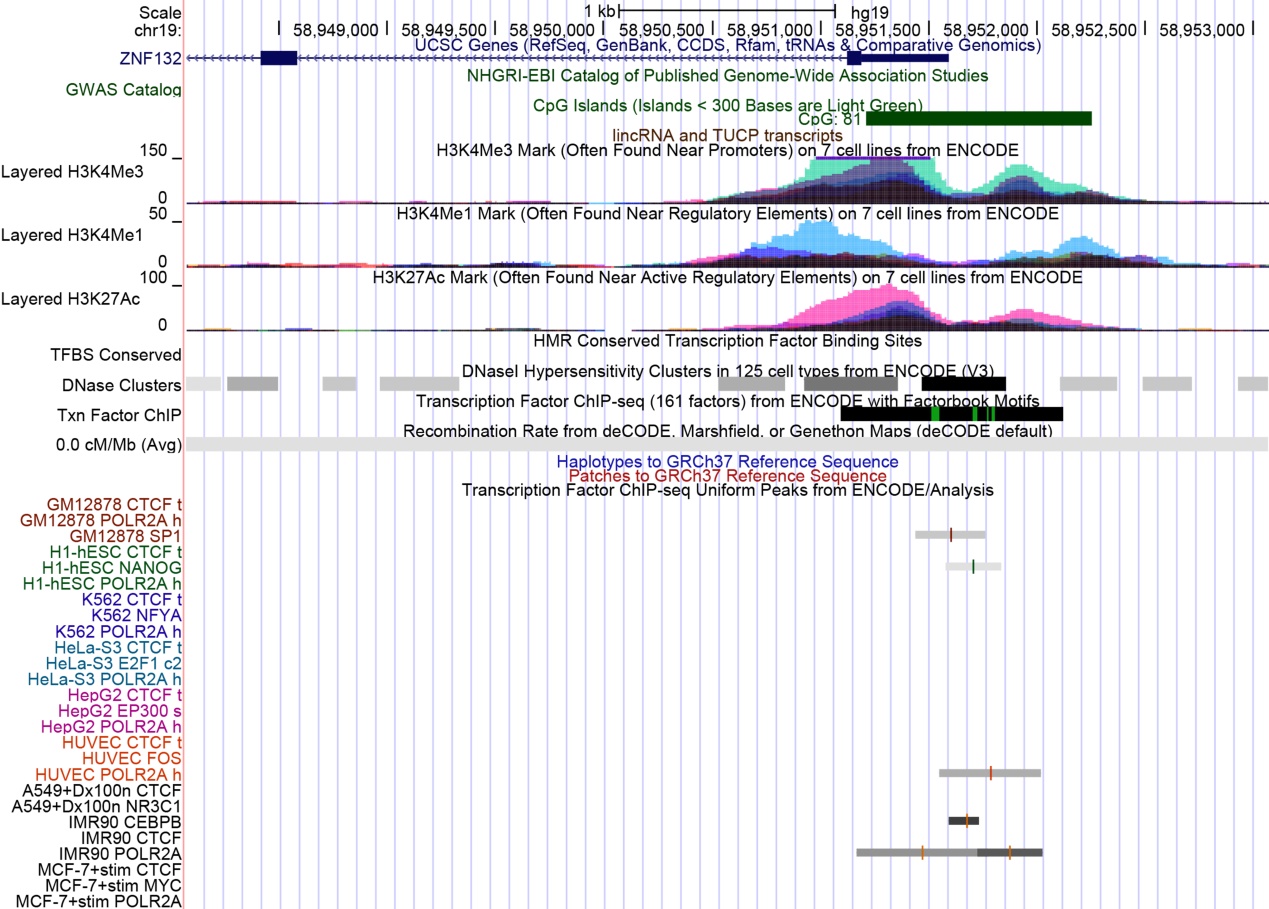


**Supplementary Figure 1**. ENCODE ChIP-seq data to show Sp1 bindings in CpG-island of *ZNF132* promoter region. Integrated Regulation from ENCODE Tracks based on hg19 was captured from UCSC Genome Browses. Transcription Factor ChIP-seq (161 factors) from ENCODE with Factorbook Motifs  Data version: ENCODE Mar 2012 Freeze was used in this study.
