# Supplementary material for: Epigenetic silencing of ZNF132 mediated by methylation-sensitive Sp1 binding promotes cancer progression in esophageal squamous cell carcinoma
Source: Cell Death Dis. 2018 Dec 18;10(1):1. doi: 10.1038/s41419-018-1236-z (PMC6315024; doi:10.1038/s41419-018-1236-z)
Supplement: Supplementary file 2 — Supplementary Table 1 [file 41419_2018_1236_MOESM2_ESM.docx]

**Supplementary Table 1：Sequences of primers used in this study**

| Primer | Target | Sequence, 5’-3’ | Use |
| --- | --- | --- | --- |
| *ZNF132* F | Coding region | GTCATTGAGAGGCGGGACT | qPCR |
| *ZNF132* R | Coding region | TCGGGAACACCTTGGCTCAT | qPCR |
| *ZNF132* Xba I | CDS sequence | GCTCTAGAATGGCCCTGCCCAGC | PCR |
| *ZNF132* Not I | CDS sequence | ATAAGAATGCGGCCGCTCAGGTATGAATCTT | PCR |
| GAPDH F | Coding region | GAAGGTGAAGGTCGGAGTC | qPCR |
| GAPDH R | Coding region | GAAGATGGTGATGGGATTTC | qPCR |
| *ZNF132* F | Promoter region | GGTGTTTTAGGGTTGGTTATTGG | BSP |
| *ZNF132* R | Promoter region | TACCTTCCTCRCTCCTATTTCCATAA | BSP |
| LINE-1 F | Positive control | AGTAGGGYGAGGTATTGTTTTATTTG | BSP |
| LINE-1 R | Positive control | AAACTACTATACTAACAATCAACARAATTCC | BSP |
| ChrM F | Negtive control | TGTGTGGAAAGTGGTTGTGTAGATATT | BSP |
| ChrM R | Negtive control | AATCACAAATCTATCACCCTATTAACCA | BSP |
